# Age and gender specific normal values of left ventricular mass, volume and function for gradient echo magnetic resonance imaging: a cross sectional study

**DOI:** 10.1186/1471-2342-9-2

**Published:** 2009-01-21

**Authors:** Peter A Cain, Ragnhild Ahl, Erik Hedstrom, Martin Ugander, Ase Allansdotter-Johnsson, Peter Friberg, Hakan Arheden

**Affiliations:** 1 Dept of Clinical Physiology, Lund University Hospital, SE-22185, Lund, Sweden; 2 Dept of Radiology, Sahlgrenska Academy, SE-413 45, Gothenburg, Sweden; 3 Dept of Clinical Physiology, Sahlgrenska Academy, SE-413 45, Gothenburg, Sweden

## Abstract

**Background:**

Knowledge about age-specific normal values for left ventricular mass (LVM), end-diastolic volume (EDV), end-systolic volume (ESV), stroke volume (SV) and ejection fraction (EF) by cardiac magnetic resonance imaging (CMR) is of importance to differentiate between health and disease and to assess the severity of disease. The aims of the study were to determine age and gender specific normal reference values and to explore the normal physiological variation of these parameters from adolescence to late adulthood, in a cross sectional study.

**Methods:**

Gradient echo CMR was performed at 1.5 T in 96 healthy volunteers (11–81 years, 50 male). Gender-specific analysis of parameters was undertaken in both absolute values and adjusted for body surface area (BSA).

**Results:**

Age and gender specific normal ranges for LV volumes, mass and function are presented from the second through the eighth decade of life. LVM, ESV and EDV rose during adolescence and declined in adulthood. SV and EF decreased with age. Compared to adult females, adult males had higher BSA-adjusted values of EDV (p = 0.006) and ESV (p < 0.001), similar SV (p = 0.51) and lower EF (p = 0.014). No gender differences were seen in the youngest, 11–15 year, age range.

**Conclusion:**

LV volumes, mass and function vary over a broad age range in healthy individuals. LV volumes and mass both rise in adolescence and decline with age. EF showed a rapid decline in adolescence compared to changes throughout adulthood. These findings demonstrate the need for age and gender specific normal ranges for clinical use.

## Background

Fundamental structural and functional properties of the left ventricle including left ventricular mass (LVM), volumes, and function are often assessed in the clinical setting using two dimensional echocardiography. The clinical use of cardiac magnetic resonance imaging (CMR) has increased lately and a consensus panel report has established clinical indications for cardiovascular magnetic resonance [1].

It is of great clinical importance to be able to differentiate between normal and abnormal findings, but this may often prove difficult if adequate normal values are unknown. The need for age and gender specific normal values with CMR is therefore growing. Several studies have defined CMR normal ranges of LV volumes and function in limited age ranges [2-8], and none of these have examined these parameters over a wide age range in healthy individuals. A recent study presented age and gender specific normal ranges for CMR at 1.5 T using a steady state free precession sequence [9]. However, at times, some centers still need to use gradient echo sequences in clinical assessment at 1.5 T.

The aim of the study was, therefore, to suggest clinically usable age and gender specific normal ranges for LV volumes and function using gradient echo CMR at 1.5 T for the second through the eighth decade of life. We also sought to explore, in a cross sectional study, the normal variation of LV volumes and function in strictly healthy subjects over a wide age range in order to examine the age variation of these parameters.

## Methods

### Study population and design

The study population consisted of 96 healthy volunteers prospectively recruited by advertisement from the local community (76 adults, age 21–81 and 20 children, age 11–15, all caucasian). No subjects were excluded because of poor image quality. All subjects had a normal electrocardiogram (ECG) and blood pressure (systolic blood pressure (SBP) ≤ 140 mmHg and diastolic blood pressure (DBP) ≤ 90 mmHg) [10] and had no history of systolic or diastolic hypertension. Subjects with previous or current cardiovascular, systemic, metabolic disease, body mass index ≥ 30, visually overt aortic or mitral valve regurgitation in long axis MR images, or treatment with medication (except oral contraceptives [n=5], hormone replacement therapy [n=5] or oral incontinence medication [n=1]) were excluded from the study. MR imaging was performed within four weeks after inclusion with image analysis undertaken by independent observers blinded to subject characteristics. The investigation protocol and procedures were approved by the Lund University research ethics committee (reference number LU 207-00). Written informed consent was obtained from all subjects prior to inclusion.

### Blood pressure

SBP and DBP were obtained in the supine or seated position by auscultation using a brachial cuff.

### MR imaging

All patients were imaged in the supine position using a 1.5 T system (Magnetom Vision; Siemens, Erlangen, Germany) with a 25 mT/m gradient system and a phased-array body coil. Standard scout images were used to locate the orthogonal planes of the heart. End-expiratory ECG triggered short-axis gradient echo cine loops were then acquired throughout the left ventricle from the base (atrioventricular valve plane) to the apex. Typical imaging parameters were TR = 100 ms, and echo sharing gave an effective phase interval of 50 ms, TE = 4.8 ms, slice thickness 10 mm, field of view 350–420 mm, matrix 126 × 256, flip angle 20°. The number of cardiac phases per acquisition was determined as the integer obtained from the RR interval divided by TR. Nine to twelve slices were required to completely cover the left ventricle, depending on heart size.

#### MR image analysis

##### i) Tracing of endocardial and epicardial contours

**Figure 1:**
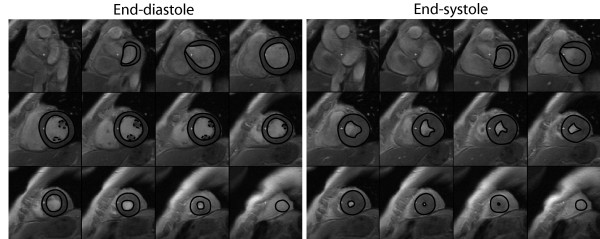
**Delineation of the left ventricular borders in the short axis plane in end-diastole and end-systole**. Both the endocardial and epicardial borders were outlined manually for both ventricular volume and mass measurement (solid lines). Papillary muscle measurements were only included for mass measurements (dashed lines).

All measurements were undertaken manually without the aid of automated image analysis software. End-diastolic and end-systolic frames were identified according to ventricular blood pool area. Measurements of the left ventricular endocardial and epicardial areas (Scion Image Beta 4.0.2, Scion Imaging Corporation) in each image frame were performed in the short-axis view. At the base of the left ventricle, the aortic outflow tract below the valve was included in volume measurements. The free papillary muscles were included for LVM assessment, and excluded for left ventricular volume assessment [11]. In the basal region of the heart where the left atrium was seen, only the portion of the slice that could be identified as left ventricle was included for measurement (Figure 1).

##### ii) Left ventricular mass and dimensions

The difference in area between the endocardial and epicardial contours multiplied by the slice thickness (10 mm) represented myocardial volume for a given slice. Total myocardial mass was obtained by calculating the sum of all myocardial slice volumes and multiplying by the myocardial specific gravity (1.05 g/cm^3^). End-diastolic volume (EDV) and end-systolic volume (ESV) were calculated as the endocardial volume at end-diastole and end-systole respectively. Stroke volume (SV) was calculated as the difference between EDV and ESV. Ejection fraction (EF) was calculated as SV divided by EDV. All parameters were divided by body surface area (BSA) to achieve values adjusted for body size (e.g. LVM/BSA for left ventricular mass adjusted for BSA). In addition, a subset of 20 subjects were analyzed by 2 readers blinded to each others results in order to examine interobserver variability. Intraobserver variability was assessed by one observer who performed repeated blinded measurements in the same 20 subjects one month later.

### Statistical analysis

SPSS (version 16) was used for statistical calculations. A *p *value less than 0.05 was considered statistically significant. Values are expressed as mean ± SD. Unpaired Student's t test or ANOVA were used to test for significance between groups since both visual inspection and the Kolmogorov-Smirnov test showed that all measures were adequately normally distributed. Intra- and interobserver variability were assessed as the mean difference of measurements ± SD and by the intraclass correlation coeffecient (ICC) employing a two-way mixed model [12]. The coefficient of variation (CV) was calculated as the SD of the difference between two measurements expressed as percent of their mean. The coeffiecient of repeatability (CR) was calculated as two times the SD of the difference in two measurements. Pearson's correlation coefficient was used to assess the correlation between two variables, and expressed as its square (R^2^). BSA was calculated using a previously described technique [13]. Curve estimation and the 95% prediction intervals of LVM, dimensions, and function were defined using commercial software (Matlab curve fitting toolbox, Matlab version R12, Mathworks). The most appropriate curve fitting algorithm for LVM, dimensions and functional measures was identified as the rational polynomial of the form P(x)/Q(x) where both numerator and denominator were at most of the second degree and which had the highest adjusted R^2 ^and lowest root mean square (RMS) of the error [14]. The predicted lower, mean and upper limits for normal values of LV parameters in each decade were calculated as the average of the mean and 95% prediction interval of the predicted values for each whole year as given by the curve estimation model.

## Results

### Population description

Data from 94 of the 96 subjects have, in part, previously been published in a study of LVM and wall stress [14]. Table 1 displays the baseline characteristics for the current study population. Blood pressure was similar between genders and well within accepted normal limits [10].

**Table 1:** Baseline characteristics of the adult and adolescent study populations according to gender (mean ± SD).

	Adults			Adolescents		
	Male	Female	p	Male	Female	p

Number	41	35		9	11	

Age (years)	45 ± 16	46 ± 17	0.70	13 ± 2	12 ± 1	0.22

Height (m)	1.81 ± 0.07	1.68 ± 0.06	<0.001	1.63 ± 0.13	1.59 ± 0.08	0.46

Weight (kg)	80 ± 10	64 ± 10	<0.001	55 ± 14	50 ± 6	0.33

BSA (m^2^)	2.01 ± 0.15	1.73 ± 0.15	<0.001	1.56 ± 0.26	1.48 ± 0.13	0.38

BMI (kg/m^2^)	24.4 ± 2.3	22.7 ± 2.9	0.006	20.2 ± 2.6	19.7 ± 1.9	0.58

SBP (mmHg)	123 ± 9	123 ± 11	0.84	108 ± 10	108 ± 9	0.97

DBP (mmHg)	74 ± 7	72 ± 8	0.36	61 ± 3	61 ± 8	0.85

BMI = body mass index, BSA = body surface area,

DBP = diastolic blood pressure, SBP = systolic blood pressure.

### Normal variation of left ventricular measures with age

Table 2 lists suggested age-specific reference values for LV mass, EDV, ESV, SV and EF as absolute values and adjusted for BSA for males and females, respectively.

**Table 2:** The predicted lower, mean and upper limits for normal left ventricular parameters in males and females of different ages.

	LVM (g)			LVM/BSA (g/m^2^)			EDV (ml)			EDV/BSA (ml/m^2^)			ESV (ml)			ESV/BSA (ml/m^2^)			SV (ml)			SV/BSA (ml/m^2^)			EF (%)		
Age (y)	Lower	Mean	Upper	Lower	Mean	Upper	Lower	Mean	Upper	Lower	Mean	Upper	Lower	Mean	Upper	Lower	Mean	Upper	Lower	Mean	Upper	Lower	Mean	Upper	Lower	Mean	Upper
MALES																											
11–20	108	152	197	64	87	110	84	138	192	53	78	104	12	45	79	9	26	42	58	94	129	35	52	70	52	67	82
21–30	150	193	235	73	95	118	115	167	219	58	82	107	32	64	96	16	32	48	68	102	137	33	51	68	51	66	81
31–40	154	196	238	74	96	119	113	165	217	57	81	105	35	67	99	17	33	49	64	98	132	31	48	65	51	65	80
41–50	149	191	233	73	95	117	105	156	208	53	77	102	33	65	97	16	32	48	57	91	125	29	46	63	50	65	79
51–60	141	183	225	70	92	114	94	145	197	48	73	97	29	61	93	14	30	46	51	85	119	26	43	60	50	64	79
61–70	130	173	216	66	89	111	80	133	185	43	67	92	23	55	88	12	28	44	43	78	112	22	39	57	49	64	78
71–80	118	163	207	61	85	108	65	120	174	36	62	88	15	49	83	8	26	43	35	71	106	16	36	55	49	63	78
																											
FEMALES																											
11–20	80	125	171	56	79	101	77	120	162	55	76	96	13	37	60	10	22	34	53	84	116	36	53	71	55	71	86
21–30	98	142	186	60	82	104	76	119	161	52	72	93	17	40	63	12	24	35	49	81	112	32	49	66	55	70	86
31–40	98	142	186	59	81	103	75	118	160	49	70	90	19	42	64	12	24	36	46	77	108	29	46	63	54	70	85
41–50	96	140	183	58	80	102	74	116	158	47	68	88	20	43	65	13	25	36	43	74	105	26	43	60	54	69	85
51–60	93	136	180	57	79	101	73	115	158	46	66	87	21	44	67	13	25	37	40	71	103	24	41	58	53	69	84
61–70	88	133	177	55	77	100	72	114	157	45	66	86	22	45	68	14	26	38	37	69	100	23	40	57	53	69	84
71–80	83	129	174	53	76	99	69	113	158	44	65	87	22	46	70	14	26	39	34	66	99	21	39	57	53	68	83

BSA = body surface area, EDV = end diastolic volume, EF = ejection fraction,

ESV = end systolic volume, LVM = left ventricular mass, SV = stroke volume.

### i) Left ventricular mass

**Figure 2:**
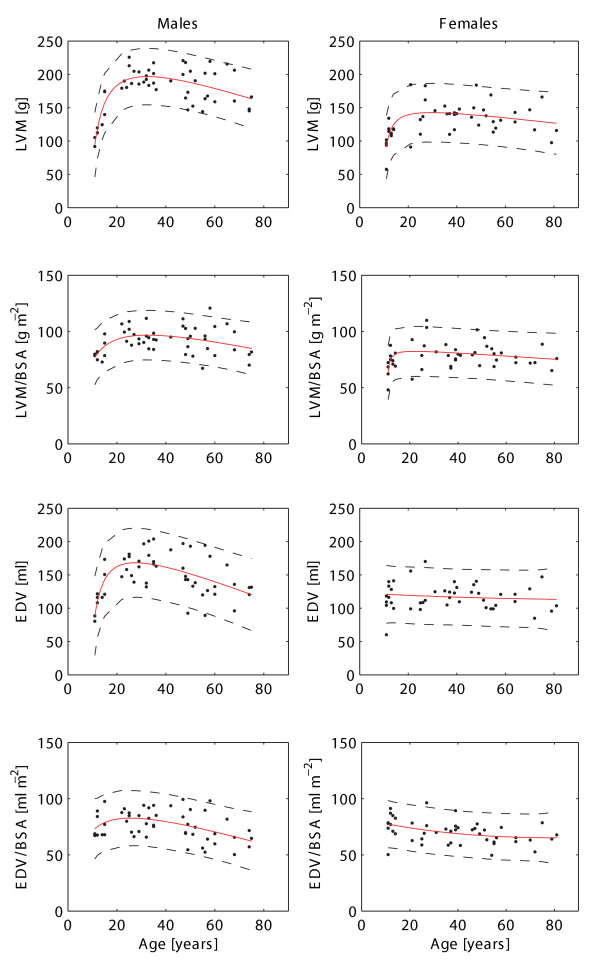
**The normal age variation in left ventricular parameters in males (left) and females (right)**. Solid lines represent rational polynomial curve fit and dashed lines the 95% prediction intervals of this fit. Reference values are listed in Table 2. LVM = left ventricular mass, BSA = body surface area, EDV = end-diastolic volume.

**Figure 3:**
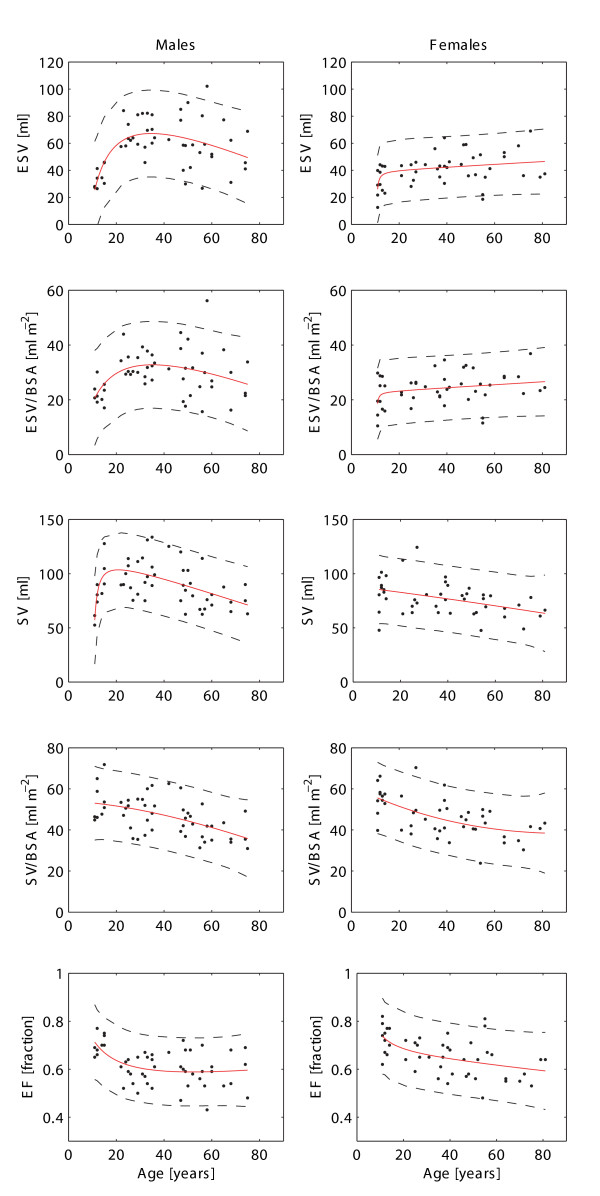
**The normal age variation in left ventricular parameters in males (left) and females (right)**. Solid lines represent rational polynomial curve fit and dashed lines the 95% prediction intervals of this fit. Reference values are listed in Table 2. BSA = body surface area, ESV = end-systolic volume, SV = stroke volume, EF = ejection fraction.

Information on the age dependence of LV mass and wall stress in the majority of this population have been published and discussed previously [14]. The current study, however, provides tabular values in order to ease the use of this information as reference values. Figure 2 and Figure 3 describe the changes in left ventricular mass with age.

### ii) End-diastolic and End-systolic volume

Ventricular volumes varied markedly between males and females (Figure 2 and Figure 3). Notably, there was a rise in EDV and ESV during adolescence and early adulthood in males, with a decline thereafter, possibly reflecting the concurrent change of LVM with age. This was absent in females with a trend towards a decrease of EDV and EDV/BSA with age and increase of ESV and ESV/BSA with age. Ventricular volumes in the adolescent group were similar between males and females (EDV: 120 ± 29 ml vs. 114 ± 23 ml, p = 0.66; EDV/BSA: 76 ± 11 ml/m^2 ^vs. 77 ± 11 ml/m^2^, p = 0.95; ESV: 35 ± 8 ml vs. 32 ± 11 ml, p = 0.48; ESV/BSA: 22 ± 4 ml/m^2 ^vs. 21 ± 7 ml/m^2^, p = 0.65). However, differences between genders became apparent for all ventricular volumes in the adult group with higher values in the male group (EDV: 153 ± 30 ml vs. 118 ± 19 ml, p < 0.001; EDV/BSA: 76 ± 13 ml/m^2 ^vs. 69 ± 9 ml/m^2^, p = 0.006; ESV: 63 ± 17 ml vs. 43 ± 11 ml, p < 0.001; ESV/BSA: 31 ± 8 ml/m^2 ^vs. 25 ± 6 ml/m^2^, p < 0.001).

### iii) Left ventricular stroke volume and ejection fraction

In general, both SV and EF demonstrated a decline with age (Figure 3). Although differences in SV and EF between males and females were present in the adult group (SV: 91 ± 19 ml vs. 75 ± 15 ml, p < 0.001; SV/BSA: 45 ± 9 ml/m^2 ^vs. 44 ± 9 ml/m^2^, p = 0.51; EF: 0.59 ± 0.07 vs. 0.64 ± 0.08, p = 0.014), SV and EF were similar between genders in the adolescent group (SV: 85 ± 22 ml vs. 83 ± 16 ml, p = 0.82; SV/BSA: 54 ± 9 ml/m^2 ^vs. 55 ± 7 ml/m^2^, p = 0.70; EF: 0.71 ± 0.04 vs. 0.73 ± 0.06, p = 0.36). Notably, left ventricular EF in the adolescent group was higher (Figure 3) compared to adult subjects (0.72 ± 0.05 vs. 0.61 ± 0.07, p < 0.001) with a decline in both males and females from ~70% to ~60% with age.

### Normal ranges for left ventricular mass, volumes and function

All variables varied according to age group (ANOVA, p < 0.05) except for EDV/BSA (p = 0.13) and ESV/BSA (p = 0.06) in males, and EDV (p = 0.39), EDV/BSA (p = 0.15), ESV/BSA (p = 0.28), SV (p = 0.14) and LVM/BSA (p = 0.24) in females.

### Intra- and interobserver variability

Data on intra- and interobserver variability are presented in Table 3.

**Table 3:** Intra- and interobserver variability

	EDV	ESV	SV	EF	LVM
**Intraobserver variability**					

Mean difference ± SD, absolute	0 ± 4 ml	-1 ± 10 ml	1 ± 8 ml	1 ± 6%	1 ± 3 g

Mean difference ± SD, relative	1 ± 5%	-1 ± 5%	1 ± 6%	1 ± 4% of EF	1 ± 5%

Coefficient of variance	4%	10%	8%	6%	3%

Coefficient of repeatability	8 ml	20 ml	16 ml	12%	6 g

R^2^	0.96	0.87	0.85	0.47	0.97

Intra-class correlation coefficient	0.99	0.96	0.96	0.81	0.99

					

**Interobserver variability**					

Mean difference ± SD, absolute	3 ± 4 ml	-5 ± 14 ml	8 ± 7 ml	4 ± 6%	1 ± 5 g

Mean difference ± SD, relative	5 ± 6%	-2 ± 6%	6 ± 6%	3 ± 4% of EF	2 ± 9%

Coefficient of variance	4%	12%	7%	7%	6%

Coefficient of repeatability	8 ml	28 ml	14 ml	12%	10 g

R^2^	0.96	0.85	0.82	0.44	0.91

Intra-class correlation coefficient	0.98	0.95	0.91	0.81	0.98

## Discussion

This study suggests age and gender specific normal values for LV mass, volumes and function measured in a healthy population over a large age range using gradient echo CMR at 1.5 T.

One of the most important tasks in patient examination is to distinguish normal findings from those indicative of disease. Normal ranges for a given parameter encompassing 95% of the population constitute the mainstay of this procedure. With increasing use of cardiac magnetic resonance imaging, the need for age-specific ranges of LV mass, volumes and function for this modality is obvious. This is of specific importance in order to correctly exclude disease.

### A) Left ventricular mass

The findings regarding left ventricular mass in the present study and its relation to earlier studies has been discussed earlier [14]. Other non-invasive techniques including three dimensional echocardiography [15,16] and computerized tomography [17] have overcome many of the shortcomings of two dimensional echocardiography although no reference values for normal patients have been established in a large population.

### B) Left ventricular volumes, stroke volume and ejection fractions

Six CMR studies [3-5,7-9] have described overall values of EDV, ESV, SV, and EF, which are consistent with findings from other imaging modalities and are, in the corresponding age ranges, broadly consistent with the findings in this study, with one exception. Sandstede *et al *used a gradient echo sequence and reported as much as 20–30% lower values for LV mass and volumes. Those authors chose to only measure LV volumes and mass in short axis slices which contained more than 50% of the circumference of the LV wall in *both *end-diastole and end-systole. This approach does not take into account the long axis movement of the basal parts of the left ventricle [11], which in part may explain the lower values obtained in that study. Hudsmith *et al*, showed differences between subjects above and below the age of 35 [8], but continuous age-specific values of these parameters, however, have not been described using gradient echo CMR in populations that extend over the breadth of age as in the present study. We show that adult females had a slight progressive decrease in ESV with age, with a resulting slight progressive increase in EF, whereas Maceira *et al *[9], showed the opposite. BMI and blood pressure were similar for both populations, hence it is likely some other factor which contributes to the difference between the studies. The discrepancy is difficult to interpret, and one can only speculate as to what difference between the populations may explain this discrepancy.

The inotropy of the left ventricle has been shown to be related to growth hormone/IGF levels [18]. Although our study has not measured growth hormone levels, one might speculate that the higher EDV, SV and EF in younger subjects may possibly, in part, reflect the higher growth hormone levels within this age group. Alternatively, these higher values may be due to physical activity rather than hormones. However, we have previously published that self-reported physical activity increased with age in our population [14]. Thus, it is unclear exactly why these measures are larger in younger subjects.

### Reproducibility of measurement

The reproducibility of CMR LV measures obtained in our study was consistent with previously reported studies of intra- and interobserver variability [19-21].

### Limitations

Regurgitation in the aortic or mitral valve was only assessed visually in long axis CMR images, and this is a limitation. However, it is likely that this was sufficient to serve the purposes of this study in this population with otherwise unremarkable ECG, medical history and physical examination. The number of included subjects in each gender and decade is less than the minimally suggested n = 10 [21] for the size of a group needed to determine reference values. However, the suggestion of n = 10 is based on the use of the mean +/- 2SD of those subjects' measurements as a basis for calculating reference values. In contrast, we used a curve estimation model and its 95% prediction interval to determine our reference values. Using this method, the robustness of the reference values is based on data from the entire population and less susceptible to small numbers of subjects in individual decade groups. The normal values provided in this study are appropriate for studies undertaken using similar gradient echo sequences at 1.5 T. It has been shown that steady-state free precession based sequences may result in slightly lower LVM and greater LV volumes [7,22,23]. These differences are likely to be systematic in nature and do not alter the physiological significance of the age trends reported in the current study. Furthermore, the use of steady-state free precession based sequences at a field strength of 3 T is currently hampered by artifacts [24]. The presented normal values for gradient echo sequences at 1.5 T may thereby also be of value for assessing results from gradient echo cardiac imaging at 3 T.

## Conclusion

This study suggests normal reference values for left ventricular mass, dimensions, and function in healthy humans from early adolescence to the eighth decade according to gender using gradient echo CMR at 1.5 T. LV volumes, mass and function vary over a broad age range in healthy individuals. LV volumes and mass both rise in adolescence and decline with age. EF showed a rapid decline in adolescence compared to changes throughout adulthood. These findings further demonstrate the need for age and gender specific normal ranges for clinical cardiac MR examinations.

## Abbreviations

(BSA): body surface area; (CMR): cardiac magnetic resonance; (DBP):diastolic blood pressure; (ECG): electrocardiogram; (EDV):end-diastolic volume; (EF): ejection fraction; (ESV): end-systolic volume; (ICC): intraclass correlation coefficient; (LV): left ventricle; (LVM): left ventricular mass; (SBP): systolic blood pressure; (SV): stroke volume; (T): Tesla; (TE): time to echo; (TR): time to repetition.

## Competing interests

The authors declare that they have no competing interests.

## Authors' contributions

PC performed data acquisition, data analysis, statistical analysis and drafted the manuscript. RA and AAJ performed data acquisition and data analysis, and participated in critically revising the manuscript for important intellectual content. EH performed data acquisition, data analysis, statistical analysis and helped to draft the manuscript. MU performed data acquisition, data analysis, statistical analysis and helped to draft the manuscript. PF conceived of the study, participated in its design and coordination, and participated in critically revising the manuscript for important intellectual content. HA conceived of the study, participated in its design and coordination, and helped to draft the manuscript. All authors' read and approved the final manuscript.

## Pre-publication history

The pre-publication history for this paper can be accessed here:

http://www.biomedcentral.com/1471-2342/9/2/prepub
